# Expansion of seasonal influenza vaccination in the Americas

**DOI:** 10.1186/1471-2458-9-361

**Published:** 2009-09-24

**Authors:** Alba María Ropero-Álvarez, Hannah J Kurtis, M Carolina Danovaro-Holliday, Cuauhtémoc Ruiz-Matus, Jon K Andrus

**Affiliations:** 1 Comprehensive Family Immunization Project, Pan American Health Organization, 525 Twenty Third St., NW, Washington DC 20037-2895, USA

## Abstract

**Background:**

Seasonal influenza is a viral disease whose annual epidemics are estimated to cause three to five million cases of severe illness and 250,000 to 500,000 deaths worldwide. Vaccination is the main strategy for primary prevention.

**Methods:**

To assess the status of influenza vaccination in the Americas, influenza vaccination data reported to the Pan American Health Organization (PAHO) through 2008 were analyzed.

**Results:**

Thirty-five countries and territories administered influenza vaccine in their public health sector, compared to 13 countries in 2004. Targeted risk groups varied. Sixteen countries reported coverage among older adults, ranging from 21% to 100%; coverage data were not available for most countries and targeted populations. Some tropical countries used the Northern Hemisphere vaccine formulation and others used the Southern Hemisphere vaccine formulation. In 2008, approximately 166.3 million doses of seasonal influenza vaccine were purchased in the Americas; 30 of 35 countries procured their vaccine through PAHO's Revolving Fund.

**Conclusion:**

Since 2004 there has been rapid uptake of seasonal influenza vaccine in the Americas. Challenges to fully implement influenza vaccination remain, including difficulties measuring coverage rates, variable vaccine uptake, and limited surveillance and effectiveness data to guide decisions regarding vaccine formulation and timing, especially in tropical countries.

## Background

Influenza is a highly infectious viral disease transmitted through respiratory droplets. Annual epidemics are estimated to cause between three to five million cases of severe illness and 250,000 to 500,000 deaths worldwide [1,2]. Annual vaccination remains the main strategy for primary prevention [3]. Seasonal influenza vaccines are safe and provide a cost-effective tool to reduce the disease burden [2,3]. The vaccine is composed of two influenza type A viruses and one type B virus. Due to the constant risk of antigenic drift-minor point mutations to the viral genome-vaccines are reconfigured annually for both the Northern and Southern Hemispheres [2]; the effectiveness of the resulting vaccine depends on the degree of match between vaccine viruses and circulating strains. Among healthy populations under 65 years of age, the seasonal influenza vaccine is 70-90% effective at preventing illness. Among the elderly, the vaccine is 30-40% effective at preventing disease, but 50-60% effective at preventing hospitalization, and 80% effective at preventing death [4].

Decisions regarding annual vaccine composition for both hemispheres are made by the World Health Organization (WHO) based on viral surveillance data from WHO's Global Influenza Surveillance Network (FluNet) [5]. The use of either the Northern or Southern Hemisphere vaccine depends on the pattern and timing of seasonal influenza circulation. The epidemiology of influenza in temperate regions of the Americas is well-described. In these regions the peak influenza season occurs during the cold winter months: November-March in the Northern Hemisphere and April-September in the Southern Hemisphere [6]. The epidemiology of seasonal influenza in tropical countries, however, remains less well defined. In the tropics, influenza transmission does not correspond to distinct peaks, but is thought to occur on a year-round basis with epidemics typically occurring between the seasons in the Northern and Southern Hemispheres [7].

In the Americas, Bermuda, Canada, Chile, and the United States have utilized seasonal influenza vaccination for the past several decades. Most other countries and territories in Latin America and the Caribbean had not introduced the vaccine into the public health sector until fairly recently. In Latin America and the Caribbean, vaccines offered through the public sector are purchased through governmental funds and available to the public free of charge. In 2003, during the 56^th ^World Health Assembly, the World Health Organization (WHO) recommended nations to increase seasonal influenza vaccination coverage in all their high-risk groups. WHO posited the goal of achieving 50% vaccination coverage in populations 65 years and older by 2006 and 75% coverage in this population by 2010 [2,8]. In 2004, the Pan American Health Organization's (PAHO) Technical Advisory Group on Vaccine-preventable Diseases (TAG) expanded recommendations for the Americas. PAHO's TAG is comprised of eight immunization and vaccine experts that meet biennially, in the presence of Member States' immunization representatives, to provide recommendations on vaccination policy and strategies to improve countries' vaccination efforts [9]. TAG recommended yearly seasonal influenza vaccination for populations older than 60 years, chronically ill individuals, immunodeficient individuals, health professionals, and pregnant women in their second trimester. In 2006, PAHO's TAG further expanded routine vaccination to include children aged 6-23 months [10].

The goal of this paper is to describe the status of seasonal influenza vaccination in the Americas through December 2008, with a focus on Latin America and the Caribbean, and to discuss future challenges for optimizing use of this vaccine in the Region.

## Methods

Influenza data reported by countries to the Comprehensive Family Immunization Project (IM) at PAHO headquarters through December 2008 were compiled and analyzed. Data were retrieved from seven sources: a PAHO survey administered by IM to Member States in 2004, the 2006 WHO Global Influenza Survey, annual country reports through the PAHO-WHO/UNICEF Joint Reporting Forms (JRF), information from PAHO's Revolving Fund for Vaccine Procurement (RF) [11], publications of governmental public health authorities [12,13], communications with PAHO immunization focal points in country offices, and a 2008 seasonal influenza questionnaire (see additional file 1). For the purpose of this exercise, information from the five islands of the Netherlands Antilles was based on data received from the island of Bonaire. Data from the French Departments in the Caribbean were not included in this exercise as they do not routinely report to PAHO.

The following information was collected: year of vaccine introduction into the public and private sectors, vaccine formulation used, timing of annual vaccination campaigns, purchase of vaccine through the RF, criteria used for vaccine introduction, the status of vaccine impact evaluations, population risk groups targeted for vaccination, and the associated coverage rates. Vaccination coverage rates in most Latin American and Caribbean countries are calculated using administrative data, dividing the number of doses of vaccine administered in the target age group by the census projection for that group.

## Results

**Figure 1:**
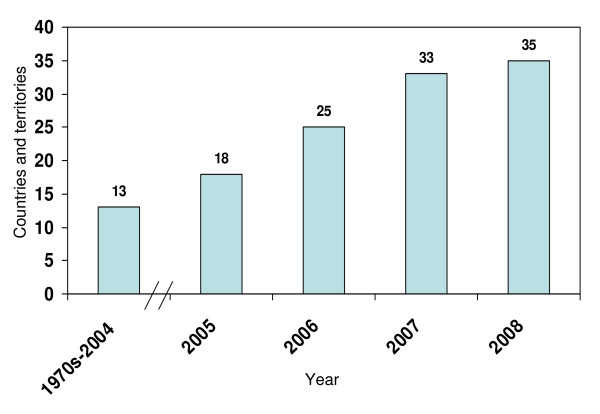
**Number of countries and territories in the Americas with public policies regarding seasonal influenza vaccination (1970s-2008)**. *Note*: Data not collected from the French Departments (French Guiana, Guadeloupe, and Martinique). **Source: Country and territory reports to PAHO**.

Until 2004, 13 countries and territories had introduced the seasonal influenza vaccine into their public health systems; of these countries, only Bermuda, Canada, Chile, and the United States had been using the vaccine for multiple decades (Table 1). Every year subsequent to 2004, substantial increases in the uptake of the seasonal influenza vaccine have been observed. In 2005, five additional countries and territories introduced the vaccine, followed by seven, eight, and then two countries and territories in 2006, 2007, and 2008, respectively. As of December of 2008, 35 out of the 43 countries and territories in the Americas included in this analysis had incorporated the vaccine into their public health systems (Figure 1). The use of the seasonal influenza vaccine through the private sector in the Region, in some cases many years prior to its introduction into the public sector, has been widespread, but not quantified.

**Table 1:** Year of seasonal influenza vaccine introduction and population groups vaccinated in the Region of the Americas, 2008.

				**Coverage in older adults (%)**			**Other Risk Groups**				
**Country**	**Year of Vaccine Introduction**	**Children**	**Older Adults**	**2006**	**2007**	**2008**	**Health workers**	**Chronic diseases**	**Pregnant Women**	**Poultry Workers**	**Other**

Anguilla	2005		✓^A^				✓				

Antigua and Barbuda	2007	6-35 m	≥ 60 y								

Argentina	1993	6-23 m^A^	≥ 65 y	50			✓	✓	✓	✓	✓^B^

Aruba	NA										

Bahamas	2005	6 m-5 y	≥ 65 y				✓	✓	✓		

Barbados	2006		✓^C^				✓				✓^D^

Belize	2008	6 m-23 m	≥ 65 y				✓	✓			

Bermuda	1970s	6 m-18 y	≥ 60 y	65^E^	60		✓	✓	✓		✓^F^

Bolivia	NA										

Brazil	1999		≥ 60 y	85.7	86.6	86.9	✓	✓			✓^G^

British Virgin Islands	2007	> 3 y	≥ 65 y				✓	✓^H^			✓^I^

Canada	1970s	6-23 m	≥ 65 y				✓	✓	✓	✓	✓^J^

Cayman Islands	1990	6-23 m	≥ 50 y				✓	✓	✓		✓^K^

Chile	1975	6-23 m	≥ 60 y	89^L^	88.6	89.1	✓	✓	✓	✓	✓^M^

Colombia	2005	6-23 m	≥ 65 y				✓	✓			

Costa Rica	2004	6 m-8 y^A^	≥ 65 y				✓	✓			

Cuba	1998	< 24 y^N^	≥ 65 y	100	100		✓	✓		✓	

Dominica	NA										

Dominican Republic	2006	6-23 m	≥ 50 y				✓	✓		✓	✓^O^

Ecuador	2006	6-23 m	≥ 65 y	67	57.5		✓				

El Salvador	2004	6-23 m	≥ 60 y	99	92	100	✓	✓			

Grenada	2007	6 m-5 y	≥ 60 y				✓			✓	

Guatemala	2007		≥ 60 y^P^		100		✓				

Guyana	NA										

Haiti	NA										

Honduras	2003	> 6 m^A^	≥ 60 y^Q^	90	83		✓			✓	

Jamaica	2006	6 m-5 y^A^	≥ 60 y^A^				✓				

Mexico	2004	6-35 m, 3-9 y^A^	≥ 60 y	93.3	84.9		✓	✓			

Montserrat	2007	< 9 y					✓				✓^R^

Netherlands Antilles (Bonaire)	2007	✓^A^	≥ 65 y	100	100	100		✓			

Nicaragua	2007	6-23 m^A^	≥ 65 y^A^								

Panama	2005	6-23 m	≥ 60 y	86.2	79		✓	✓		✓	

Paraguay	2005	6-23 m	≥ 60 y	74	73		✓	✓		✓	

Peru	2008						✓				

St. Kitts	NA										

St. Lucia	2006		≥ 65 y				✓	✓			

St. Vincent	NA										

Suriname	NA										

Trinidad and Tobago	2007	6-23 m	≥ 60 y				✓	✓		✓	✓^S^

Turks and Caicos	2006	6 m-5 y	≥ 60 y				✓	✓			

USA	1940s	6 m-18 y	≥ 50 y	36 (50-64 y) 65.6 (≥ 65 y)^T^			✓	✓	✓		✓^U^

Uruguay	1996	6-23 m, > 23 m^A^	≥ 65 y	29.2	31.1		✓	✓		✓	

Venezuela	2006	6-23 m	≥ 60 y	61.9	20.6		✓	✓			

**Source: Country and territory reports to PAHO**.

A. With chronic disease; B. Essential services, security forces, and educators; C. The elderly in institutions; D. Military and front line staff; E. Coverage in 2006 is for populations ≥ 65 y; F. Others includes children on aspirin therapy, essential workers, travelers, close contacts or caregivers of individuals with chronic disease, and individuals living in crowded conditions; G. Indigenous population including population > 6 m and incarcerated populations; H. Renal dysfunction. I. Residents of nursing homes; J. Residents of nursing homes, those in contact with populations at high risk for influenza complications and those who provide essential community services; anyone else aged 2-64 years should be encouraged to get vaccinated; K. Police and fire, children < 6 m who are at high risk for complications and individuals caring for babies < 6 months; L. Those older than 65; M. Egg producers; N. With asthma/diabetes; O. Public safety workers; P. In institutions; Q. ≥ 60 y in 2008 and ≥ 65 y in 2006 and 2007; R. High risk workers; S. National security; T. Coverage data are from the National Health Interview Survey (NHIS) for the 2006-2007 season; U. People in contact with those at high risk and all persons who want to reduce the risk of becoming ill with influenza or of transmitting it to others.

Countries and territories in the Americas target a wide range of risk groups in their seasonal influenza vaccination recommendations. As of the end of 2008, 33 countries and territories were targeting older adults; of these, Barbados and Guatemala targeted only those individuals living in institutions. Anguilla, Jamaica, and Nicaragua targeted older adults with chronic disease. Thirty-two countries and territories targeted health workers. Twenty-four countries and territories targeted individuals with chronic disease, such as lung disease, cardiovascular diseases, metabolic diseases, renal dysfunction, and immunosuppressant diseases.

Twenty-nine countries and territories targeted children for vaccination. Of these, 13 countries and territories recommended vaccinating children aged 6-23 months; two countries and territories recommended vaccinating children aged 6-35 months; one territory recommended vaccinating children older than three years; three countries and territories recommended targeting children aged 6 months-5 years; one territory recommended vaccinating children aged up to nine years; Bermuda and the United States recommended targeting all children aged 6 months-18 years [13,14]. Argentina, Bonaire, Costa Rica, Cuba, Honduras, Jamaica, and Nicaragua recommended only targeting children with chronic disease.

Additional risk groups identified for prioritized influenza vaccination included public safety workers, indigenous populations, incarcerated individuals, and childcare providers (Table 1). Eleven countries and territories also included poultry workers as targeted risk groups, while seven countries and territories included pregnant women (2^nd ^trimester) in their vaccination campaigns.

**Figure 2:**
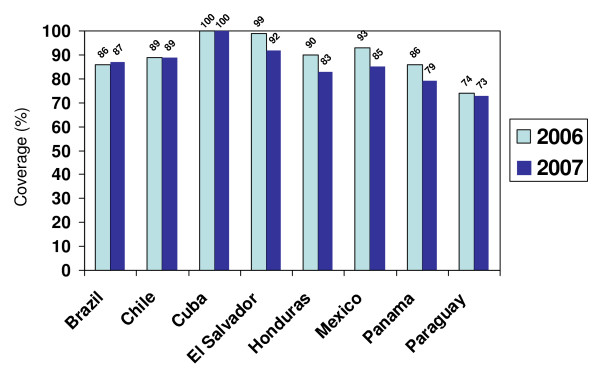
**Reported 2006 and 2007 seasonal influenza vaccination coverage among elderly populations in selected countries in Latin America**. *Note*: ≥ 65 years in Cuba, Chile (2006), Honduras; ≥ 60 years in Brazil, Chile (2007), El Salvador, Mexico, Panama, and Paraguay. **Source: Country and territory reports to PAHO**.

**Figure 3:**
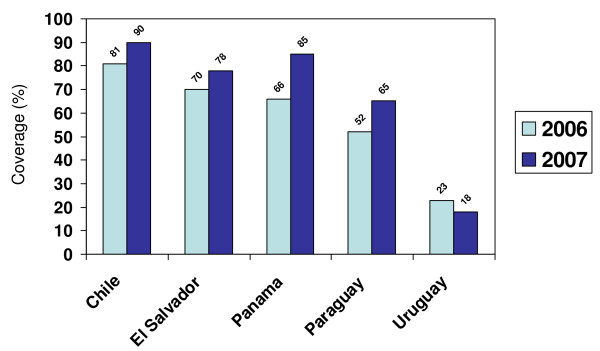
**Reported 2006 and 2007 seasonal influenza vaccine coverage among children aged 6-23 months in selected countries in Latin America**. Source: Country and territory reports to PAHO.

Consistent seasonal influenza vaccine coverage data were not widely available for most risk groups in the Region; however, 2006 and 2007 coverage data for elderly adults and children aged 6-23 months in selected countries can be found in Figures 2 and 3. Of the 14 countries that reported 2007 seasonal influenza vaccine coverage for the elderly, 12 had surpassed WHO's target of 50% coverage by 2006 and nine have already reached WHO's target of 75% coverage by 2010.

**Figure 4:**
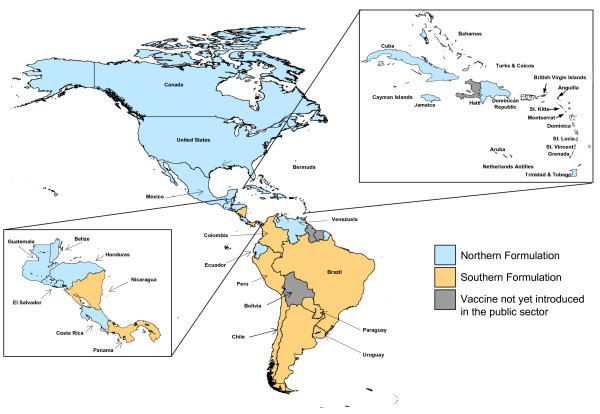
**Formulation of seasonal influenza vaccine used in countries and territories of the Americas, 2008**. Source: Country and territory reports to PAHO.

Twenty-six countries and territories utilized the Northern Hemisphere formulation of influenza vaccine in their yearly activities, whereas nine countries and territories administered the Southern Hemisphere formulation. In tropical areas of the Americas, both formulations were used; in some cases, neighboring countries administered different hemispheric formulations (Figure 4). Of note, in Nicaragua, the Southern Hemisphere formulation became available through the public sector beginning in 2007. However, the Northern Hemisphere formulation is still administered in the private sector, where it has been utilized since 2005. Colombia initially introduced the Northern Hemisphere formulation into the public health sector in Bogotá in 2005. Since 2007, the country has administered the Southern Hemisphere formulation nationwide. This decision was made based on the timing of disease peaks and the most recent vaccine formulation available.

Countries and territories in the Americas identified several criteria used to justify seasonal influenza vaccine introduction. Seven countries and territories (Belize, Bonaire, Brazil, Ecuador, Montserrat, Nicaragua, and Paraguay) identified morbidity and mortality as the main criteria influencing vaccine introduction. The Cayman Islands also identified morbidity and mortality as criteria, in addition to recommendations from PAHO and the United States' Centers for Disease Control and Prevention (CDC). Five countries (Argentina, Cuba, the Dominican Republic, Grenada, and Venezuela) specified political decisions as the influential force. In some countries and territories, vaccine introduction was attributed to a combination of factors. Eight countries (Chile, El Salvador, Guatemala, Honduras, Mexico, Panama, Peru, and Uruguay) referenced morbidity and mortality in combination with political decisions, and Colombia cited these two factors plus cost-effectiveness studies as the rationale behind vaccine introduction. In Costa Rica, seasonal influenza vaccine administration began in 2004 after a cost-effectiveness study was completed [15]. Of note, Barbados indicated that the seasonal influenza vaccine was introduced among health workers and front line staff in preparation for pandemic influenza; however, uptake among health workers has been poor. For 2009, six countries reported plans to conduct national evaluations of the impact of the seasonal influenza vaccine.

**Figure 5:**
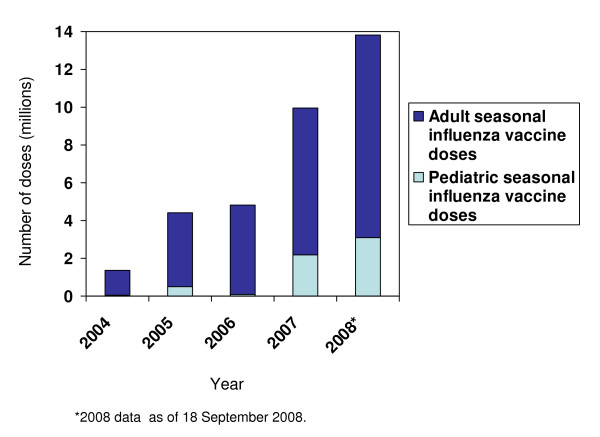
**Doses of adult and pediatric seasonal influenza vaccine purchased through the PAHO Revolving Fund (RF) for Vaccine Procurement by year**. *Note*: Brazil, Canada, Chile, Mexico, and the United States do not procure vaccine through the RF. **Source: Country and territory reports to PAHO**.

Of the 35 countries and territories that used seasonal influenza vaccine in 2008, 30 purchased the vaccine through PAHO's RF (Figure 5). The RF is a mechanism for bulk purchase of vaccines and immunization supplies, managed by PAHO since 1979, to serve Member States [11,16]. In 2004, approximately 1.4 million doses of seasonal influenza vaccine were purchased by countries through the RF; in 2008 this figure had increased to approximately 14.4 million doses (Figure 5). Outside of the RF, in 2008, Brazil, Canada, Chile, Mexico, and the United States purchased approximately 152.3 million doses of seasonal influenza vaccine for a total of approximately 166.3 million doses of seasonal influenza vaccine used in the Americas last year.

## Discussion

As of December 2008, 35 of the 43 countries and territories of the Americas were recommending seasonal influenza vaccination in the public sector, up from just 13 in 2004, representing a rapid uptake of the vaccine over the last five years. Global progress outside the Region should also be noted. In 2005, a study of 56 countries reported rapid growth in influenza vaccination in the prior decade, most notable in nations outside of North America and Western Europe [17].

In this analysis, countries and territories identified the morbidity and mortality caused by seasonal influenza and the decision to introduce the vaccine at a political level as the two most frequent reasons for seasonal influenza vaccine introduction. While an in-depth examination of other factors influencing vaccine uptake was out of the scope of this study, it is likely that the fairly rapid uptake of influenza vaccine in the Region has been multi-factorial. Fear of an influenza pandemic, the increased use of the seasonal influenza vaccine in the private sector, the actions of neighboring countries, and the influence of PAHO's TAG recommendations likely played a role in country and territory decisions to begin to administer the vaccine in the public sector.

The groups targeted for seasonal influenza vaccination varied between countries of the Americas, ranging from only health workers and/or institutionalized older adults in selected countries, to multiple groups at risk, such as persons with chronic illness, children, pregnant women, health care workers, and poultry and egg farmers. The majority of those countries reporting influenza vaccine coverage data among the elderly had surpassed WHO's targets. Selected countries likely achieved such success through large scale intensive national vaccination campaigns, carried out over two to four week time periods.

Despite the selected coverage achievements in the Region, efforts to improve influenza coverage monitoring among all targeted populations will be essential to determine what population groups are not being reached and to create vaccination strategies to ensure better compliance. Currently, in many countries, coverage rates for all targeted population groups are not routinely available. Particular challenges exist when measuring coverage with the vaccine, even among children. First, obtaining accurate numerator and denominator data to calculate coverage rates among target populations is often difficult as doses administered in the public sector are more likely to be recorded and consolidated then those given in the private sector. Even in developed countries, there are difficulties in obtaining definitive coverage figures [13,18]. Additionally, for children, in order to build sufficient immunity, individuals under nine years of age being vaccinated for the first time need to receive two doses of the seasonal influenza vaccine separated by at least a month [13]. This requirement challenges the current health systems capacity to calculate coverage data, as nationwide individualized vaccination registries are not common and most countries in Latin America and the Caribbean rely on administrative data to calculate coverage.

Uptake of the seasonal influenza vaccine across all groups at risk also needs to be improved. The seasonal influenza vaccine is unique compared to other vaccines. To be effective, it must be administered annually, primarily through time-limited campaigns, and target population groups that are not necessarily accustomed to vaccination. Furthermore, factors such as the public perception of influenza risk and vaccine effectiveness may vary by year, affecting vaccine uptake.

There was consistent use of the Northern or Southern Hemisphere vaccine formulations in temperate countries of the Americas, with the timing of vaccine campaigns dependent on the annual vaccine production process. Sufficient understanding of the viral circulation patterns in temperate countries of the Region made the decisions about vaccine formulation clear and uniform across national borders. In contrast, there was a mix of formulations and timing of annual campaigns in tropical countries. This may reflect the limited information available regarding the epidemiology of influenza in tropical areas. Recent studies have illustrated how seasonal influenza epidemics appear to be impacted by the introduction of new viral strains into a population [6], and how the initiation of influenza A epidemics seem to follow latitudinal gradients, moving from more tropical regions towards the poles as the season progresses [6,19]. Nevertheless, in order to better understand viral circulation in tropical areas of the Americas, influenza viral surveillance needs to be enhanced. Having better surveillance data will facilitate decisions regarding the optimal timing of vaccination campaigns and the best vaccine formulation to use. Of note, since the advent of a Southern Hemisphere vaccine formulation in 1999, this formulation has matched the prior season's Northern Hemisphere formulation in 5 out of eleven years. Advances in viral surveillance in tropical areas will help avoid the current reliance on information generated from isolated studies. In the Americas, work in this area has already begun through a cooperative agreement between PAHO and CDC to strengthen influenza surveillance in the Region [20]. For children, some countries in the Americas are integrating sentinel influenza surveillance with existing pneumonia surveillance systems.

Countries that have not introduced the seasonal influenza vaccine would benefit from evaluating local influenza epidemiology and conducting cost-effectiveness studies in order to develop the most informed, evidence-based policies. To this end, PAHO's ProVac initiative has been established to help countries conduct such evaluations to make evidence-based decisions [21]. Countries already administering the seasonal influenza vaccine may benefit from evaluating the impact of the vaccine's use and, more specifically, the formulation administered. Such research would be especially informative if completed in large countries, such as Brazil, that utilize one vaccine formulation, but have both tropical and subtropical zones and varying influenza disease peaks [19]. Studies in Brazil suggest that the Southern Hemisphere vaccine formulation, currently utilized nationwide, may not have a significant impact in the reduction of morbidity and mortality among elderly populations in northern areas of the country (Brazil Ministry of Health unpublished data). Disease cases in Northern Brazil peak from March to May; ideally vaccination here would occur in February, which is prior to the availability of the Southern Hemisphere vaccine formulation [22]. Studies analyzing the impact of using the vaccine formulation available prior to disease peaks (Northern Hemisphere) in these areas would be of interest. Furthermore, research in neighboring countries with similar climates, but in which different vaccine formulations are currently administered, would also be informative.

The annual supply of seasonal influenza vaccine needs to meet the accelerated demand. In order to increase production capacity for seasonal influenza vaccine in the Americas, Brazil and Mexico are currently in the process of establishing influenza vaccine production facilities through technology transfer agreements brokered through the WHO [23]. This will be particularly important as countries expand their recommended populations groups and age ranges targeted for seasonal influenza vaccination, as the United States has done with the recommended age for childhood vaccination, moving from six months-23 months to six months-five years in 2006, and to six months-18 years in 2008 [13,14].

This paper aims to provide a general overview of the use of seasonal influenza vaccine in the Region of the Americas. This study has several limitations, including the following: data were limited for some countries and territories and information sources did not provide in-depth evaluation of the specific reasons taken for vaccine introduction. Additionally, as is done for most reporting of vaccine coverage rates for childhood diseases, seasonal influenza coverage rates reported were calculated using administrative data, which have inherent limitations. The main findings, however, are solid: while some countries in the Americas have been utilizing seasonal influenza vaccine for many decades, a rapid uptake of the vaccine has occurred in the last five years, the targeted groups vary, there is a mix of formulations used in tropical areas, and coverage data is limited and may need to be further validated. In-depth studies to understand the factors for vaccine introduction in more detail would also be useful.

## Conclusion

Since 2004 there has been rapid uptake of seasonal influenza vaccine in the Americas. However, countries continue to face challenges to fully implement influenza vaccination in populations at risk. Strategies are needed to expand vaccination uptake, improve coverage monitoring, and enhance surveillance. Effectiveness evaluations will be crucial to better understand the impact of seasonal influenza vaccination in countries of the Americas.

## Competing interests

The authors declare that they have no competing interests.

## Authors' contributions

AMRA, HJK, and MCDH were the main writers of the manuscript. HJK gathered and summarized the data and AMRA, HJK, and MCDH all contributed to the literature review, analysis and interpretation of the data and the conception of the manuscript. JKA and CRM played important roles in critically revising the manuscript as well as drafting and implementing policy. All authors read and approved the final manuscript.

## Pre-publication history

The pre-publication history for this paper can be accessed here:



## Supplementary Material

Additional file 1 **Questionnaire sent to national authorities in 2008 to update information regarding seasonal influenza vaccination**. The file provided shows an image of the English version of the 2008 seasonal influenza questionnaire which was sent to countries and territories in the Americas. A Spanish version of the questionnaire was also elaborated. This questionnaire was one of the sources of information utilized in this article. Click here for file
