# Supplementary figures and images for: Expansion of seasonal influenza vaccination in the Americas
Source: BMC Public Health. 2009 Sep 24;9:361. doi: 10.1186/1471-2458-9-361 (PMC2764707; doi:10.1186/1471-2458-9-361)

## Slide 1
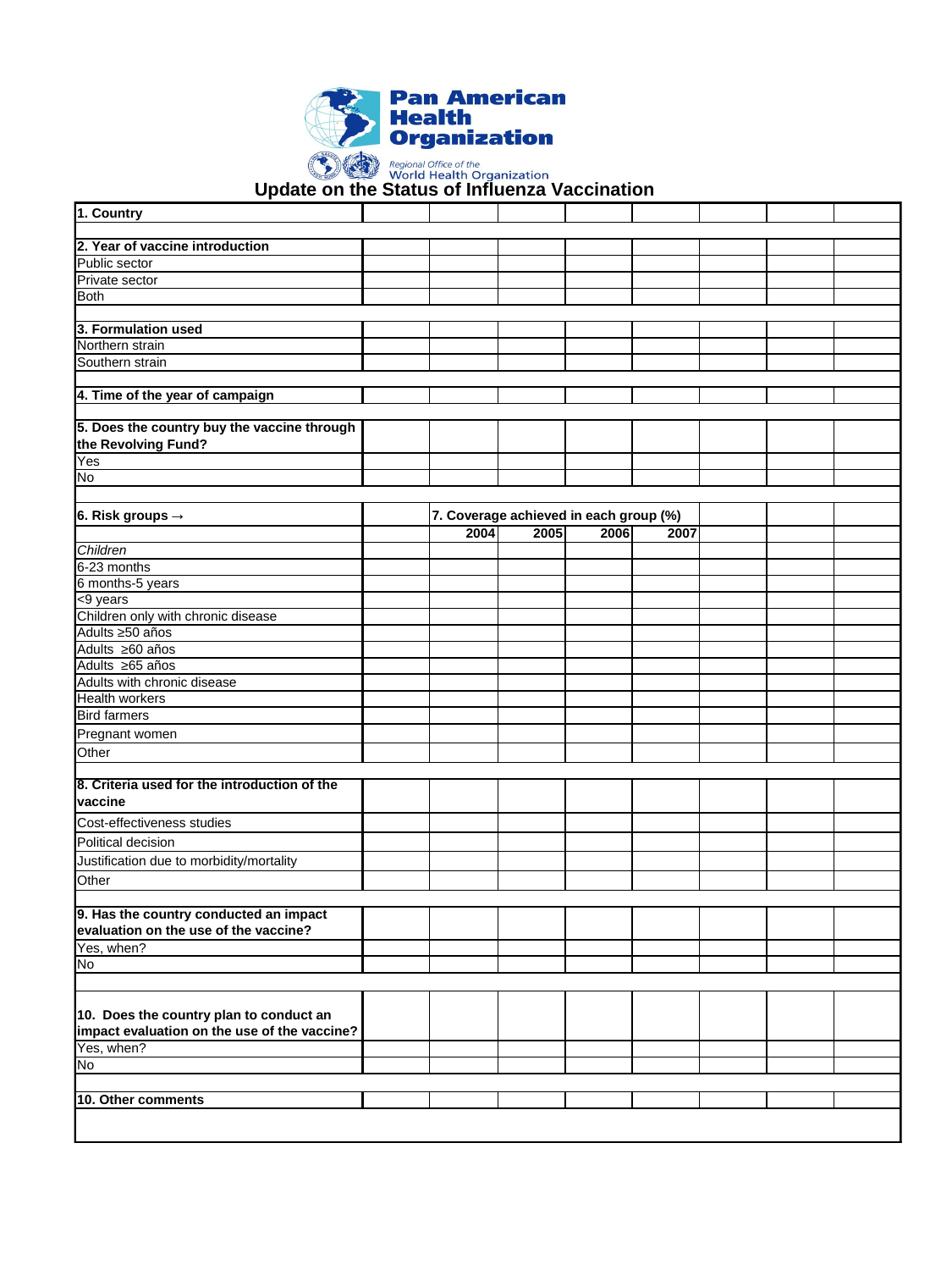

Supplement: Additional file 1 — Questionnaire sent to national authorities in 2008 to update information regarding seasonal influenza vaccination. The file provided shows an image of the English version of the 2008 seasonal influenza questionnaire which was sent to countries and territories in the Americas. A Spanish version of the questionnaire was also elaborated. This questionnaire was one of the sources of information utilized in this article. [file 1471-2458-9-361-S1.PPT]
